# Pregnancy postponement and childlessness leads to chronic hypervascularity of the breasts and cancer risk

**DOI:** 10.1038/sj.bjc.6600600

**Published:** 2002-11-12

**Authors:** H W Simpson, C S McArdle, W D George, K Griffiths, A Turkes, A W Pauson

**Affiliations:** University Department of Surgery, Glasgow Royal Infirmary, Glasgow G31 2ER, UK; University Department of Surgery, Glasgow Royal Infirmary, GlasgowG12 8QQ, UK; University of Wales, College of Medicine, Cardiff, CF3 2UR, UK; CRW Research Lab, Velindre Hospital, Cardiff CF14 2TL, UK; Glasgow University, 7 Merrycrest Avenue, Glasgow G46 6BY, UK

**Keywords:** breast, physiology, reproductive variables, cancer risk and epidemiology, chronobra

## Abstract

Epidemiologists have established that women with small families, and particularly nulliparae, are prone to develop breast cancer later in life. We report that physiological mammary hypervascularity may be an intermediate reason against the background that breast-core vascularity is normal in pregnancy but pathological in the vascularisation of cancer. We examined breast ‘core’ vascularity in nulliparae during their potential reproductive life and in parous women after their last birth but before their menopause. Fifty clinically normal pre-menopausal non-pregnant women (100 breasts) were studied daily for one ‘luteal positive’ menstrual cycle. Their parity history varied from zero to five babies. Under controlled domestic conditions each wore a special electronic thermometric bra to automatically record breast ‘core’ temperature changes as a measure of mammary tissue blood flow. In the nulliparae there was a rise of breast vascularity throughout reproductive life. In the parous women, a year or so after each birth, breast vascularity was reset at a lower level than before the pregnancy; thereafter, as in nulliparae, there was progressive increase in mammary vascularity until the menopause.

*British Journal of Cancer* (2002) **87**, 1246–1252. doi:10.1038/sj.bjc.6600600 www.bjcancer.com

© 2002 Cancer Research UK

## 

Reproductive factors continue to be highlighted in epidemiological studies of breast cancer. Nuns and other nulliparous women have long been known to have a higher risk of breast cancer than the average. Fairly recently a large Swedish study concluded that each additional birth conferred a 10% risk reduction (Lambe *et al*, 1996). Very recently a large cohort study in France reported again that low parity is associated with increased risk of breast cancer (Clavel-Chapelon, 2002).

Here we investigate the relationship between parity and long-term breast-core blood flow. This is relevant since carcinogenesis is dependent on a blood supply from the host (Hanihan and Folkman, 1996). Our observations have been carried out daily on 50 women (100 breasts) over one menstrual cycle. The studies were at various times *post menarche* in nulliparae, and at various times *post partum* in pre-menopausal parous women. The studies were possible because an electronic thermometric bra was available designed to measure breast-core temperatures and to store the readings in real time: the so-called Chronobra (Simpson and Green, 1977).

A physiologist might have expected that the lack of function in the breasts of nulliparae or in non-breast-feeding parous women would be associated with a low mammary-core blood flow. Herein we provide evidence that the reverse is the case. We document there is a progressive increase of the vascularity in breasts of women of reproductive age in the intervals before, between and after pregnancies. Cumulatively, this is particularly marked in the childless.

We suggest that this ‘paradox’ may in part explain the increased cancer risk in women of low parity.

## MATERIALS AND METHODS

The thermometric data represent an estimated measurement of physiological breast ‘core’ blood flow changes over one menstrual cycle (Simpson and Green, 1977) by the thermometric Chronobra. The temperatures represent an Internal Bioassay of mammary tissue vascularity.

The thermometric bra (Simpson *et al*, 1993) consists of a substantial traditional fabric garment with approximately 1 cm of air and foam–fabric insulation between the breast skin, the outside of the standard over-clothing, and the observation room ambient temperature at 20–24°C. Each cup of the bra has a seven temperature sensor array connected to a small instrument in a pouch in front (Simpson *et al*, 1993). This is all electronic including clock-initiated sampling and a memory for up to 4096 temperatures recorded in ‘real’ time. In each case the instruments were calibrated in a water bath against a British Standards thermometer using specially designed software. We considered the readings to be accurate to 0.05°C. The subject was sedentary during the daily 1.5 hour-long measurement, e.g. watching television. Characteristically, temperatures levelled off after three-quarters of an hour and the computer-identified crest was taken as the temperature for that day. These measurements are in contrast to classical infrared thermometry (Gautherie and Gross, 1980), which defines a surface distribution of temperature, i.e. the venous architecture. However, this distinction is not as sharp as it might seem, because enlargement of these veins is a classic sign of pregnancy and the veins arise from the mammary tissue.

Whereas the thermometric Chronobra measures temperatures to 1/20th°C accuracy on the breast surface, the important physio logical inference is that changes in temperature are presently considered to reflect changes in the mammary core tissue vascularity for the following reasons: first, the two breast cups of the instrument contain substantial external thermal insulation due to the thickness of the two layers of cellular fabric, the air gap between them and the standard T-shirt overclothing. This arrangement minimises outward heat flow from the breast to the environment so that after three-quarters of an hour of equlibration, seen as a levelling off of temperature in the recorded data, there is a minimal heat flow outwards and the recorded surface temperature measured reflects the temperature in the breast ‘core’. This is the principal of the so-called deep body thermometer (Fox *et al*, 1973) used in the assessment of hypothermia patients in the clinic. Second, occasionally, the ‘core’ breast temperatures recorded in practice are in excess of 37°C, i.e. blood heat. Third, whereas the dermal vascular plexuses will also exhibit a menstrual cycle of temperature due to the basal body temperature periodicity, nevertheless if the basal body temperature is subtracted from the Chronobra temperature, then a superposed ‘breast specific’ menstrual rhythm is revealed. This is documented (Simpson *et al*, 1995); consistent is that the timing of this menstrual cycle is similar to that of anecdotal measurements of breast blood flow (Simpson *et al*, 2000). Finally, the order of the menstrual cycle temperature changes, observed here on non-pregnant, non-lactating women, are consistent with vascular phenomena. We know of no other credible physiological source for such changes.

Fifty women of reproductive age and of parity history 0–5 participated in the trial. The field studies were carried out by a senior research nursing sister. Many controls were recruited from her friends, colleagues and acquaintances, also by the authors lecturing at church mothers groups, and 11 out of 50 of the subjects came from breast clinics. These latter subjects had clinically normal breasts but attended because of a family history, worry, etc. Bias from this subset was thought to be small because comparison with the remaining menstrual sets revealed only a small difference in averages. Some patients were a follow-up after fibroadenoma removal. Again their data appeared to be homogeneous with the balance and this condition is not documented as associated with increased risk (Wang and Fentiman, 1985). No cases with diffuse lumpy breasts (benign disease) were included here, i.e. the fibro-adenosis/fibrocystic disease complex.

**Figure 1:**
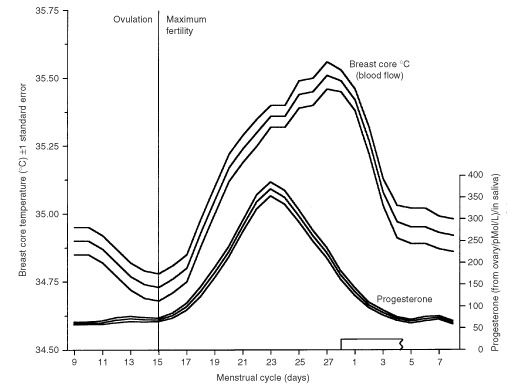
50 women of reproductive age and parity 0–5. Each subject's menstrual cycle of data represented. Right and left breast temperature averaged for each daily reading. The 50 daily readings are here subjected to a 3-day smooth with one standard error. The graphed daily points therefore represent 50×3 readings. Complementary saliva progesterone concentrations estimated daily are also given. Note that the breast core-temperatures and the progesterone values rise progressively from ovulation, but whereas breast core-temperatures peak on day 23 of a notional 28-day menstrual cycle, the breast core-temperature is maximum at the start of the menses. The synchronisation of the menstrual cycle across the subjects into a notional 28-day cycle is discussed in Methods.

Each subject self-studied both breasts daily for one menstrual cycle so as to obtain the average of 28×2 daily readings, depending on the length of her particular cycle. The average from the right and left breast for each individual was also averaged so the data for each subject was truncated into one datum. The subjects were asked to commence daily Chronobra measurements when their menses had stopped and to continue until the next menses had stopped. Cycle lengths averaged 28.4 days with a range of 23–36 days. All the data were fitted to a notional 28 days by the following procedure. The daily progesterone concentration in saliva enabled the peak of the luteal phase to be identified. From the literature (Presser, 1974) we deduced that on the average 28-day cycle this peak occurred on day 23 (see also Dyrenfurth *et al*, 1974). For each individual this calendar date was called ‘day 23’, and the phasing was obtained by counting up to 28 and down to 21 from this point. For those with a cycle longer than 28 days, e.g. 36 days, the initial 8 days of breast temperature of the cycle were omitted from the calculation of the menstrual average. For those with a short cycle, e.g. 23 days, there was a ‘wrap around’ from the beginning of the next cycle to fill up the missing 5 days to obtain the 28-day average. The appropriateness and effect of these procedures can be gauged from Figure 1

in the Results section, where the averaged menstrual cycles for the 50 women are plotted with their standard errors.

In all instances we also obtained the middle line (the so-called Mesor) of a tau=28-day cosine function fitted by least squares to the 28 data. In the averaging procedure, missing data were filled by linear interpolation. In practice, the ‘averaging’ or Mesor methods gave a very similar 28-day result. Underlying this method, as in the counting backwards from the next cycle method, is the observation that the length of the menstrual cycle consists of a fairly constant luteal phase (*c*. 12 days) and an inconstant follicular phase. Over 1000 women, of the same order of age as studied here, the standard deviation of the length of the pre-ovulatory phase was 4–5 days, whereas the luteal phase was under 2 days (see Presser, 1974, p 158).

The subject also collected a daily 1 ml saliva sample for progesterone analysis to assess the ‘luteal status’ of the particular cycle (Fahmy *et al*, 1982). She also recorded daily oral temperature measurements (so-called basal body temperature). Exclusion criteria included anovulatory cycles, fever, certain medications and more than 4 days of missing data. Regarding the oral temperature measurements, occasionally oral temperature was used to assess objectively the situation when subjects reported feeling unwell. In this instance investigators could decide whether to discontinue on the grounds of ‘fever’.

## RESULTS

### Introduction

The peak temperatures for the right and left breast were identified automatically from the daily response peaks at 45–60 min by computer software. The measurement protocol required a full menstrual cycle (e.g. 28 days) of data. One grand average temperature was obtained from these data (e.g. 28 days×2 breasts) for each subject. Overall some 12% of the data are missing for operational and technical reasons.

In this way the 50 subjects are represented here by 50×2 breast ‘core’ temperatures, in turn representing 50 menstrual cycles whose timing and luteal status had been attested by daily progesterone estimates. The data come from different women and are therefore serially independent.

### The raw data

The averaged breast-core temperatures from the 50 women (100 breasts) are shown in Figure 1. The graphed data are by day but with 3-day averaging. A highly significant menstrual rhythm of breast-core blood flow is noted. There is a lag from the progesterone data which serve to identify the luteal phase.

### Breast-core temperature and age of subject

**Figure 2:**
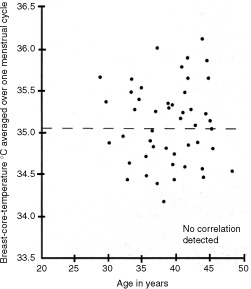
The menstrual cycle data of Figure 1 has been truncated into one point averaging the whole cycle in right and left breasts in each of the 50 subjects. Note the absence of any detected correlation between the breast core-temperature and age of the subject at study.

The 50 menstrual span ‘core’ temperatures are plotted against the ages of the subjects at the time of the study. Inspection of Figure 2

does not reveal any obvious relationship between the two variables; and when they are correlated, the coefficient is actually close to zero (*r*=0.121; *n*=50).

### Breast-core temperature and years since pregnant(or menarche in nulliparae)

**Figure 3:**
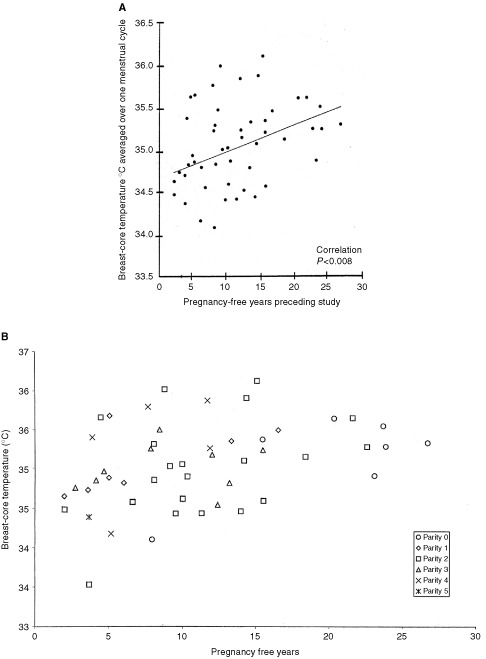
(**A**) Notice the highly significant correlation between the breast core-temperature in the 50 subjects of parity 0–5 and the pregnancy-free year interval preceding the Chronobra study. (**B**) The parity provenance of the data in (**A**).

Low-value breast-core temperatures appeared to occur particularly in subjects studied in the early post-partum years; to investigate this impression the 50 temperature data were paired with the number of pregnancy-free years immediately prior to the Chronobra study. The regression equation for the 50 pairs is as follows:

The mean breast-core temperature of right and left breast=34.7°C+0.0312×years since last pregnancy or menarche *n*=50; *t*=2.79; *P*=0.008. Figure 3

illustrates this regression line.

A highly significant positive correlation is apparent in Figure 3. There is a rise in breast-core temperature each pregnancy-free year. This finding, and the absence of correlation with age as seen in Figure 2, implies an interruption of the setting of the breast-core temperature post-partum. (Of course, there will have been extreme hypervascularity during pregnancy as part of the preparation for possible lactation but we are not concerned with that physiological change here.) In Figure 2 the random ages of the subjects at their pregnancies apparently obscures the effect seen in Figure 3a; further, the hypervascularity of nulliparae moves steadily upwards with age. The correlation in Figure 3a in which parities 0–5 are represented, prompted us to plot the parity provenances of the data.

The distribution of the parities in Figure 3(b) reveals some bias from nulliparae in the long interval pregnancy-free years; but this has to be viewed against the regression equations when the 50 data are partitioned into three subsets: nulliparae, uniparae and multiparae, all of which are rather similar.

### Multiple regression analysis

To examine further the relationship between breast-core temperature and the reproductive variables of age, parity and pregnancy-free years, a simultaneous fit was made and the results are given in Table 1

**Table 1:**
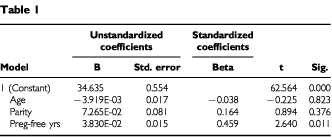

.

Of the variables studied, only pregnancy-free years are found to be associated with breast-core temperature.

### Graphical reconstruction of the post-partum resetting mechanism

The regression equations for the subsets in which single-tailed ‘P’ values are cited are as follows:

Nulliparae: breast-core°C=34.0°C+0.0571×years since menarche

*P*=0.041 (for the seven subjects)

Uniparae: breast-core°C=34.7°C+0.0490×years since pregnant

*P*=0.062 (for seven subjects)

Multiparae: breast-core°C=34.7°C+0.0370×years since pregnant

*P*=0.025 (for 36 subjects)

In Figure 4

**Figure 4:**
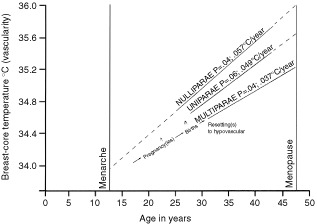
The regression equations for nulliparae, uniparae and multiparae (*n*=50 total) are given in the Results section. Here these equations are plotted using the average age of each group for the *x* axis. Note that, according to these equations, nulliparity and childlessness in general tend to higher breast vascularity.

the regression equations for these three subsets have been plotted together. The average age of each subset has been used to centre the data on the plot. The interrupted lines indicate extrapolation.

From the average ages of the uniparae and multiparae sub-populations and their regression equations we have constructed Figure 4. This illustrates that, as parity increases, there is a hierarchy of breast-core temperatures at the menopause depending on parity. Nulliparae head the hierarchy because the progressive vascularity has been uninterrupted by any pregnancy re-setting.

### Breast-core temperature and the interval between the last pregnancy and Chronobra study

**Figure 5:**
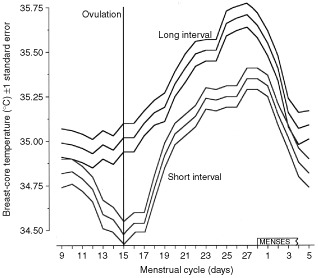
Same data as Figure 1 but in this case the data are partitioned by whether the subject had had a longer than average or shorter than average (11.2 years) pregnancy-free interval prior to the Chronobra study. As predicted from the regression equations, a long interval from the last pregnancy (or menarche in nulliparae) is associated with hypervascular breasts, a phenomenon, as the figure shows, particularly evident in the luteal phase.

The highly significant positive correlation between breast-core temperature and pregnancy free years led us to partition the 50 data into those from women (of all parities) who had had a long interval between the last pregnancy and Chronobra studies and those who had a relatively short span; of the 50 data 27 came from women with less than 11.2 pregnancy-free years and 23 came from women with an interval longer than 11.2 years. The results of this calculation are plotted in Figure 5

, where it can be seen that a relatively long pregnancy-free interval is associated with a menstrual cycle of breast-core temperature running at a higher level, especially in the luteal phase, than the cycle of women with a relatively short interval. This is as predicted from the regression equations.

## DISCUSSION

The data from nulliparae exhibit a significant association between the breast-core temperature and the interval (years) between their menarche and the Chronobra studied menstrual cycle. These particular data also exhibit a significant association between age at study and the menstrual core-temperature.

In contrast, however, the 43 parous women exhibit no detectable association of breast-core temperature with either age at menarche or age at study.

But when breast temperature is correlated with the pregnancy-free years entering all 50 subjects, parous or not, then a highly significant association is found. We estimate that physiologically, when the breast is not part of the pregnancy/lactation event, there is a rise of 0.03°C per year of breast-core temperature, which we interpret as an increase of vascularity. It is implicit that there is a re-setting of this response after each pregnancy, otherwise there would be a positive correlation with age. The reproduction of the regression lines for the association of breast temperature in nulliparae, uniparae and multiparae is given in Figure 4, and these regression lines imply a downward re-setting of breast-core temperature after each pregnancy. Our data also indicate that the most vascular breasts are those of either nulliparae or in those with a long interval between the last pregnancy and the menopause.

At the start of potential reproductive life all women are nulliparous and will be characterised by an average pan-menstrual breast vascularity. In Figure 4 our data indicate that between pregnancies (and lactation) the vascularity state of the breast is not steady but rather increases predictably; the regression lines imply re-settings to a lower value, sometime in the post partum. Figure 5 illustrates the effect of the length of the interval on the pan-menstrual vascularity.

What is the connection between mammary vascularity and breast cancer? In carcinogenesis the blood supply of a cancer is part of the host and not the tumour. To progress beyond a few millimetres, a cancer has to have the genetic potential and phenotypic capability of inducing the host breast tissue to supply a ‘neovascularisation’. Historically this field has been reviewed by Gullino (1977) in ‘*The Natural History of Breast Cancer*’; also more specifically and contemporaneously by Folkman (1995).

The evidence that mammary hypervascularity may herald cancer is from three sources. First, epidemiologists have reported that there is a transient increase in breast cancer in the post partum after the physiological hypervascularity of pregnancy (Chie *et al*, 2000). Second, infra-red thermographers have reported that 38% of 784 breast clinic patients with asymmetric focal or global ‘hyperthermia’ of the breasts at initial examination developed ‘clinical’ cancer some years later (Gautherie and Gross, 1980). Third, we have reported (Simpson *et al*, 1995) that breast-hyperthermia is a feature of the ‘cancer-associated breast’ tissue, the name given by histopathologists to the remaining unilateral or contralateral mammary tissue after a breast cancer excision (Alpers and Wellings, 1985). Such mammary tissue contains large numbers of sub-clinical microscopic hyperplastic epithelial lesions (Simpson *et al*, 1982), and it has been shown by Jensen *et al* (1982) that these lesions, excised from resected breast specimens with the help of a dissecting microscope, are capable of effecting neovascularisation when set up as xenografts. It is also relevant that a woman with breast cancer has a six-fold risk above the normal of developing an asynchronous contralateral cancer (Simpson *et al*, 1995).

We interpret the present data along the following lines:

In nulliparae, throughout their ‘barren’ reproductive life, there is a progressive rise of mammary vascularity consistent with their particular risk of breast cancer. Indeed, this hypervascularity may contribute to the Wolffe parenchymal risk patterns noted in mammograms of nulliparae (Kaufman *et al*, 1991).

Our model is consistent with the epidemiologists' observations that increasing breast cancer risk occurs in nulliparae up to the age of 32. However, they have also observed that a first birth after this age is associated with a higher risk than nulliparae of the same age. How is this divergence from our model explained? In a previous article, ‘Genesis of breast cancer is in the pre menopause’, we have discussed in a subset the ‘age-specific’ effects of cancer risk factors. We consider that molecular carcinogenesis as opposed to clinical cancer has already occurred and the process is accelerated by pregnancy. The timing of this event will differ in nulliparae. Factors include the age-specific sensitivity of breast epithelium to carcinogenesis by X-rays or chemicals and an elapsed time for processing. (See Simpson *et al*, 1988. Also, Russo *et al*, 1979; Russo and Russo, 1980.)

Our present data are not sufficiently comprehensive to identify the point that many pregnancy-free years and the associated hypervascularity lead to a greater risk from pregnancy.

In parous women, there is a putative downward re-setting of the mammary vascularity after each birth to a point below the pre-pregnancy value; then post partum, a progressive rise until the next pregnancy or the end of reproductive life; but this rise is shorter in multiparae, and by the time the menopause is reached, their observed cumulated vascularity is less. This is consistent with the fact that the reported risk of breast cancer in parous women is proportionately less than in nulliparae.

During pregnancy-free intervals the progressive hypervascularity is especially marked in the luteal phases of the menstrual cycle (see Figure 5). Physiologically, with a lack of expected mammalian function, pregnancy and lactation, one might have expected hypovascularity as indeed occurs in the post menopause (Simpson, 1996; Figure 3b). This paradox is the more unexpected since peak luteal phase progesterone values relating to Figure 5 breast temperatures are unexpected. The progesterone concentrations for the ‘long’ interval of pregnancy-free years were 357±SEM 33, whereas those for the ‘short’ interval were 430±SEM 53. Thus the progesterones concentrations in the luteal phase were highly significantly lower when the vascularity was highest. Further discussions of this paradox is beyond the scope of this paper.

The thermometric Chronobra offers economic, self-measuring, non-invasive monitoring of the mammary ‘core’ vascularity in health and disease. Whereas numerous Classical Risk Factors are statistically linked to breast cancer, in fact they account for only a small proportion of the total disease. Here we deal with a factor, hypervascularity, which may be on the final common path to cancer (Wiseman, 2000). It has screening potential (Simpson *et al*, 1995). It is self-measured, non-invasive, objectively recorded and economic to assess. Families are becoming smaller (National Statistics, 2001). Physiological breast hypervascularity is becoming more frequent, and, predictably, breast cancer risk is becoming greater.
